# Effects of Monomeric and Oligomeric Flavanols on Kidney Function, Inflammation and Oxidative Stress in Runners: A Randomized Double-Blind Pilot Study

**DOI:** 10.3390/nu12061634

**Published:** 2020-06-01

**Authors:** Khrystyna O. Semen, Antje R. Weseler, Marcel J. W. Janssen, Marie-José Drittij-Reijnders, Jos L. M. L. le Noble, Aalt Bast

**Affiliations:** 1 Campus Venlo, Faculty of Science and Engineering, Maastricht University, 5911 BV Venlo, The Netherlands; a.bast@maastrichtuniversity.nl; 2 Department of Pharmacology and Toxicology, Faculty of Health, Medicine, and Life Sciences, Maastricht University, 6200 MD Maastricht, The Netherlands; a.weseler@maastrichtuniversity.nl (A.R.W.); mj.drittij@maastrichtuniversity.nl (M.-J.D.-R.); j.lenoble@maastrichtuniversity.nl (J.L.M.L.l.N.); 3 Department of Clinical Chemistry and Haematology, VieCuri Medical Center Noord Limburg, 5912 BL Venlo, The Netherlands; marceljanssen@viecuri.nl; 4 Department of Intensive Care, VieCuri Medical Center Noord Limburg, 5912 BL Venlo, The Netherlands

**Keywords:** ibuprofen, flavanols, inflammation, kidney function, neutrophil gelatinase-associated lipocalin, exercise

## Abstract

Nonsteroidal anti-inflammatory drugs are frequently used by athletes in order to prevent musculoskeletal pain and improve performance. In combination with strenuous exercise, they can contribute to a reduction of renal blood flow and promote development of kidney damage. We aimed to investigate whether monomeric and oligomeric flavanols (MOF) could reduce the severity of kidney injuries associated with the intake of 400-mg ibuprofen followed by the completion of a half-marathon in recreational athletes. In this double-blind, randomized study, the original MOF blend of extracts from grape seeds (*Vitis vinifera* L.) and pine bark (*Pinus pinaster* L.) or placebo were taken for 14 days preceding the ibuprofen/half-marathon. Urine samples were collected before and after the ibuprofen/half-marathon, and biomarkers of kidney injury, inflammation and oxidative stress were assessed. Intake of MOF significantly reduced the incidence of post-race hematuria (*p =* 0.0004) and lowered concentrations of interleukin (IL)-6 in the urine (*p =* 0.032). Urinary neutrophil-associated lipocalin, creatine, albumin, IL-8 and malondialdehyde tended to decrease. The supplementation with MOF in recreational runners appears to safely preserve kidney function, reduce inflammation and promote antioxidant defense during strenuous exercise and intake of a single dose of ibuprofen.

## 1. Introduction

Long-distance running is an increasingly practiced form of endurance exercise due to its beneficial effects on health and good accessibility for recreational athletes [1,2,3]. Running reduces cardiovascular risk, prevents development of type II diabetes, metabolic syndrome and neurodegenerative diseases [1,2,3]. At the same time, participation in competitive running events puts significant strain on the organs of a human body. Redistribution of cardiac output in favor of exercising muscles causes prominent reduction of blood flow to the kidney. While these adjustments are normal physiological adaptations to exercise, in some cases, they may impair renal function and even cause acute kidney injury (AKI) [4]. 

Although severe cases of AKI in runners are rather rare, transient increases in urinary kidney injury biomarkers, including creatinine, albumin, neutrophil gelatinase-associated lipocalin (NGAL) and cystatin C, were reported in ultramarathoners and marathoners [4,5,6]. Running a shorter distance, e.g., of a half-marathon, may also trigger a significant reversible decline in the glomerular filtration rate [7] and an increase in serum creatinine [8,9]. Exercise-induced changes in the pro-/antioxidant balance, damaging effects on skeletal muscles and an exaggerated inflammatory response were shown to contribute to the development of kidney damage [10,11]. Furthermore, an intake of nonsteroidal anti-inflammatory drugs (NSAIDs) has been recognized as a risk factor of AKI in runners [12,13]. NSAIDs reduce the production of vasodilatory prostaglandins in the kidneys and impair the maintenance of the perfusion pressure within glomeruli [14]. Despite these recognized hazards, an intake of NSAIDs before and during the race is common. The use has been reported by 45.9–49% of marathoners [13,15] and by 60.3–70% of the ultramarathon runners [16,17]. In a study involving approximately 47,000 half-marathon participants, the use of anti-inflammatory medications was documented in 8.3% of the race participants [12]. 

Adequate hydration is known to maintain kidney function during strenuous exercise. At the same time, it is unknown in how far nutrition and dietary supplements in particular can protect the kidney from exercise-induced stress. We therefore hypothesized that the preservation of the antioxidant and anti-inflammatory responses by a 14-day supplementation of monomeric and oligomeric flavanols (MOF) could reduce the strain on the kidneys during a half-marathon. MOF are widely present in food, such as apples, grapes, chocolate and red wine. MOF have been shown to protect (micro)vascular tissue via the stabilization of collagen and elastin [18]. While binding to these macromolecules, MOF prevent their degradation by temperature, oxidative stress, inflammation and proteinases [19]. Renoprotective effects of MOF have been observed in various kidney disease models [20,21]. Moreover, MOF are known scavengers of reactive oxygen and nitrogen species [22]. MOF have been shown to improve the bioavailability of nitric oxide and vasodilatory prostaglandins [23]. Moreover, antioxidant and anti-inflammatory effects of these compounds are commonly recognized [24,25]. In recreationally active athletes, MOF have been shown to beneficially influence exercise performances and have positive effects on the recovery indexes [26,27]. 

In the present study, we aimed to assess the effects of a 14-day supplementation with MOF extracted from the seeds of grapes (*Vitis vinifera* L.) and pine bark (*Pinus pinaster* L.) (MOF-VVPP) on an acute decline in kidney function and an increased inflammatory and prooxidant renal response in healthy recreational runners participating in a half-marathon and using a single dose of ibuprofen. 

## 2. Materials and Methods 

### 2.1. Study Design 

**Figure 1:**
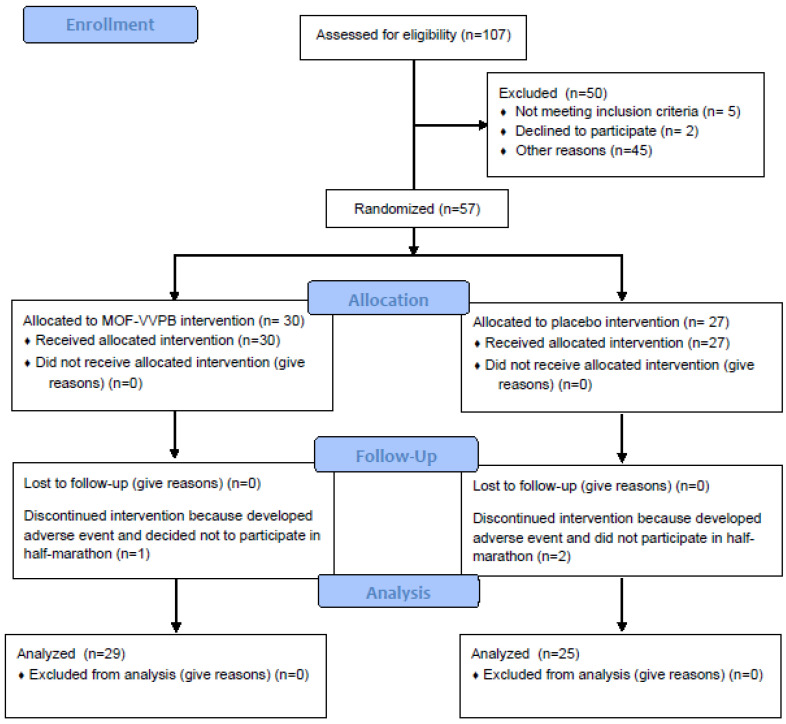
Study progress CONSORT diagram. AE, adverse events and CONSORT, Consolidated Guidelines for Reporting Trials. MOF-VVPP—monomeric and oligomeric flavanols isolated from *Vitis vinifera* L. seeds and *Pinus pinaster* L. bark.

This study was designed as a placebo-controlled, randomized, double-blind pilot trial and was conducted in accordance with the Declaration of Helsinki on biomedical research involving human subjects. The approval was obtained from the Independent Review Board Nijmegen (Nijmegen, The Netherlands, NL 65544.072.18). All participants (*n =* 57) signed the informed consent before inclusion into this study and were subsequently randomized into MOF-VVPP (*n =* 30) and a placebo group (*n =* 27). Three dropouts occurred during the 14-day supplementation period, and data from 54 participants were analyzed. Further details of the CONSORT diagram are provided in Figure 1.

### 2.2. Study Participants 

Study participants were recruited from December 2018 to March 2019 among runners preparing for the Weir Venloop half-marathon 2019. Inclusion criteria were age >18 years, at least 2 years of running experience, history of nonsteroidal anti-inflammatory drugs (NSAIDs) use and normal constant eating habits during at least 3 months prior to enrollment. Exclusion criteria included (i) history of chronic kidney or/and liver disorders, coronary artery disease, malignant hypertension, seizures, hypothyroidism and blood donation within the last 6 months; (ii) active smoking; (iii) regular intake of analgesics or drugs that influence glucose/lipid metabolism or impair renal autoregulation; (iv) vegan or vegetarian lifestyle; (v) medically prescribed or slimming diet; (vi) recent major running injuries; (vii) pregnancy and/or breastfeeding; (viii) excessive alcohol consumption; (ix) participation in another clinical trial or (half-)marathon within 4 weeks prior to inclusion; (x) use of anabolic steroids or other psychotic stimulants, addictions or other mental disorders limiting the ability to provide informed consent or to comply with the study requirements; (xi) recent (within 4 weeks before inclusion) viral or bacterial infections requiring use of antibiotics, laxatives and antidiarrheal drugs and (xii) current use of dietary supplements with renoprotective effects and potential influence on antioxidant or inflammatory status. Maintaining regular diet and training schemes, avoidance of NSAIDs, new food supplements or vitamins were requested from the runners for the duration of the study (11 March–2 April 2019). 

### 2.3. Intervention 

Participants who were allocated to the verum group received a 50/50 blend of extracts from *Vitis vinifera* L. seeds (MASQUELIER’s^®^ Original oligomeric proanthocyanidins (OPCs)) and *Pinus pinaster* L. bark (MASQUELIER’s^®^ French Pine Bark Extract; MOF-VVPP). The MOF-VVPP and placebo materials were provided by International Nutrition Company (INC) BV, Loosdrecht, The Netherlands in opaque gelatine capsules packed in bottles labeled with the treatment code. One capsule of MOF-VVPP contained 100 mg of the extracts blend, which contained catechin, epicatechin (monomeric flavan-3-ols) and oligomeric proanthocyanidins (OPCs, i.e., dimeric to pentameric flavan-3-ols; Table 1). Placebo capsules contained 100-mg microcrystalline cellulose and magnesium stearate. All participants took 2 capsules per day, which equals a daily dose of 200 mg of MOF-VVPP/placebo, for 14 days before the half-marathon. The last dose was administered in the morning before the run. Compliance was assessed based on the numbers of returned capsules as a percentage of capsules that were taken of the expected 28 capsules over the period of 14 days, and in both groups, it exceeded 95%. 

The last dose of the investigational product (200 mg) was taken in the morning approximately five hours before the start of the half-marathon (placebo, 294 ± 85 min, and MOF-VVPP, 283 ± 83 min, before the race). 

### 2.4. Study-Related Procedures

During the screening and randomization visit, potential participants were assessed for eligibility using a medical questionnaire. Heart rate, blood pressure (BP), weight and height were measured, and a urine dipstick test was performed to exclude pre-existing kidney disorders. On the day of the race, participants’ heart rate, BP and weight, as well as wellbeing and changes in concomitant medication, were assessed before and after the run. Urine samples were collected within 90 min before the race and as available within 2 h after the race. A fresh portion of urine was analyzed with a urine dipstick (2.4.). The remaining urine was centrifuged (10 min. 2000× *g*), aliquoted and stored at −80 °C for further analyses. All runners received 1 × 400 mg of oral ibuprofen (Advil Oval Tabs 400, Pfizer Inc., New York, NY, USA) 30 min before the start of the half-marathon. Intake of water and food during the race was unrestricted. Upon completion of the race, participants were asked to grade their muscle pain and feeling of fatigue by a 10-point scale with 0 being referred to as a no muscle pain/fatigue at all and 10 being maximum intensity of muscle pain or fatigue that a participant has ever experienced. 

The finishing times were obtained from the website of the Weir Venloop 2019 Two days after the run, the runners were contacted by telephone to assure that no changes in wellbeing and use of medication occurred after the race.

### 2.5. Urine Testing 

Urine dipstick test was performed with Meditape UC-10S (Sysmex Corporation, Kobe, Japan) urinalysis test strips, which allowed visual measurements of protein, blood, urobilinogen, bilirubin, ketones, glucose, nitrite, leukocytes and pH of urine. Changes in urine dipstick tests were evaluated as recommended by the manufacturer and analyzed semi-quantitatively.

Specific gravity of the urine samples was measured by refractometry (UN-4000 analyzer, Sysmex Corporation, Kobe, Japan). Albumin, creatinine, urea, uric acid and electrolyte concentrations were assessed by immunoturbidimetry; enzymatic (creatininase, urease and uricase) and indirect ion-selective electrode assays, respectively, using Cobas c6000 analyzer (Roche Diagnostics, Basel, Switzerland) with proprietary reagents. Detection limit for albumin concentration was 5 mg/L. Osmolality data were obtained with freezing-point depression osmometry. Urinary NGAL was measured with a two-step chemiluminescent immunoassay using an Architect i2000 analyzer (Abbott Diagnostics, Abbott Park, IL, USA) with a detection limit of 10 ng/mL. In cases where an uNGAL concentration was reported below that limit, the value of 10 ng/mL was used for further analysis. Cystatin C and beta-2-microglobulin were measured using the Siemens application on the platform BNII nephelometer (Siemens Healthineers, Erlangen, Germany). The detection limit for cystatin C was 0.30 mg/L and, for beta-2-microglobulin, 0.20 mg/L. 

Quantification of the lipid peroxidation end product malondialdehyde (MDA) in the urine samples was based on the formation of a fluorescent chromogen after derivatization with thiobarbituric acid [28]. The antioxidant capacity of the urine samples was quantified as trolox-equivalent antioxidant capacity (TEAC), as described previously [29], with some minor modifications and corrected for urinary uric acid concentrations quantified by HPLC (Waters Corporation, Milford, MA, USA) [30]. Concentrations of interleukin (IL)-6, IL-8, IL-18 and tumor necrosis factor (TNF)-α in the urine samples were quantified using commercially available ELISA kits (R&D Systems, Minneapolis, MN, USA). 

### 2.6. Sample Calculation

A conclusive sample size calculation for the effect of the 14-day MOF supplementation on the change in uNGAL concentrations was unfeasible due to the unknown effect size. Our unpublished observations in recreational half-marathon runners who use an NSAID before the run revealed an increase in uNGAL concentrations up to 35 ng/mL. Since we hypothesized that MOF intake will attenuate this running- and NSAID-induced increase in half-marathon runners, we estimated that a recruitment of a minimum of 44 subjects (at least 22 subjects per test group, *n* = (2 SD^2^ (Z_α_ + Z_β_)^2^)/(µ_x_ − µ_y_)^2^) will allow to detect with a power of 80% (β = 0.20; Z_β_ = 0.84) and an α-value of 0.05 (Z_α_ = 1.645) a 30% reduction in the uNGAL concentration, taking into account a standard deviation (SD) of 13.5 ng/mL. 

### 2.7. Sample Randomization 

Participants were randomly assigned to one of the two groups, i.e., the MOF-VVPP or the placebo group. Randomization was performed by a third-party (INC BV, Loosdrecht, The Netherlands) who generated the participant allocation list. 

### 2.8. Statictisal Analysis

Statistical tests have been carried out by using SPSS (version 25, IBM Corporation, Armonk, NY, USA) and GraphPad Prism software (v. 5.00, GraphPad Software, San Diego, CA, USA). Normality was assessed by the Shapiro-Wilks and Kolmogorov-Smirnov tests. For urinary parameter levels that were below the limits of detection, the lower limit values were used for the data presentation and statistical analyses. Normally distributed data were presented as mean ± SD, categorical variables as absolute numbers and population frequencies in percentage. If logarithmic transformation of data did not result in a normal distribution, data were presented as median and with the 25–75% percentiles. Changes in values were presented as mean difference and 95% confidence intervals (CI). Between-group differences were assessed with an unpaired Student’s *t*-test (normally distributed data) or the Mann-Whitney U test (data deviating from the normal distribution). Within-group differences were assessed with the Wilcoxon matched-pairs signed-rank test. The changes in the urine dipstick parameters were assessed by the exact Fisher’s test after classifying the marker as positive or negative. The level of significance was set to *p* < 0.05.

## 3. Results

### 3.1. Baseline Characteritics of the Participants 

The mean age of all participants was 47 ± 13 years and 33 (61.1%) runners were male (Table 2). On average, participants reported nine years of experience with long-distance running and trained for 36 h per week. No significant differences in the baseline characteristics between the groups were noted (Table 2). 

### 3.2. Changes in Clinical and Performance Parameters after the Half-Marathon

Body weight decreased by 0.87%, which is equal to 0.64 kg (95% CI: 0.42–0.87), in the placebo group and by 0.77%, which equals 0.55 kg (95% CI: 0.28–0.82), in the MOF-VVPP group (Table 3). This reduction in body weight after the run did not significantly differ between both groups.

After finishing the half-marathon, 11 (46%) runners in the placebo group and 20 (69%) runners in MOF-VVPP group reported pain in the leg muscles (relative risk 1.51 (95% CI: 0.91–2.48), *p* = 0.103). Furthermore, the majority of participants (20 (83%)) acknowledged being tired after the half-marathon in the placebo and 27 (97%) in the MOF-VVPP group, relative risk 1.12 (95% CI: 0.95–1.37), *p* = 0.392). The intensities of the leg muscle pain and tiredness were comparable between the groups (Table 3). The half-marathon finishing time for males and females, which characterizes running performance, did not differ significantly between the groups (Table 3).

After completion of the half-marathon, there was a significant decrease in BP in all runners, with a mean difference in systolic BP of 25.5 mm Hg (95% CI: 19.6—31.3; R^2^ = 0.772) in the placebo group vs. 29.6 mm Hg (95% CI: 20.2—38.9; R^2^ = 0.599) in the MOF-VVPP group and a mean difference in diastolic BP of 16.9 mm Hg (95% CI: 11.8—21.9; R^2^ = 0.661) in the placebo group vs. 19.0 mm Hg (95% CI: 14.0—23.9; R^2^ = 0.680) in the MOF-VVPP group. The changes in BP after the run were not statistically different in both groups (Table S1). Heart rate assessed within 40 min after completion of the half-marathon was significantly higher compared to the prerace values, with a mean difference of 15.6 bpm (95% CI: 7.6—23.5; R^2^ = 0.406) in the placebo group vs. 15.9 bpm (95% CI: 7.4—24.5; R^2^ = 0.341) in the MOF-VVPP group. Similar to the BP values, no significant differences in the heart rate between the groups were noted (Table S1). 

### 3.3. Prevalence of Hematuria and Proteinuria after the Half-Marathon

**Figure 2:**
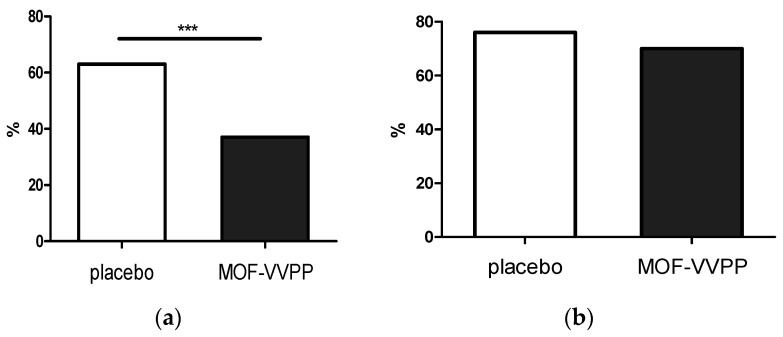
Prevalence of hematuria (**a**) and proteinuria (**b**) after the ibuprofen half-marathon challenge measured by urine dipstick in recreational runners supplemented for 14 days with 200 mg/day monomeric and oligomeric flavanols isolated from *Vitis vinifera* L. seeds and *Pinus pinaster* L. bark (MOF-VVPP). *** *p* < 0.001.

Dipstick analysis of the fresh portions of urine after the race demonstrated the presence of hemoglobin in 11 (38%) runners in the MOF-VVPP group and in 16 (64%) runners in the placebo group, *p* = 0.0004 (Figure 2). The odds of developing hematuria after running a half-marathon under 400-mg ibuprofen were 2.9 times higher in the placebo group compared to the MOF-VVPP group. Additionally, the tendency to develop higher grades of hematuria was observed in the placebo group (U = 279, z = −1.600 *p =* 0.110, r = −0.22; Table S1).

Postrace proteinuria was noted in the majority of runners 19 (76%) in the placebo group and 20 (69%) in the MOF-VVPP group without significant differences in the severity of this symptom between the groups (U = 308, z = −0.996, *p* = 0.319, r = −0.135). Presence of leukocytes, urobilinogen and ketones were shown in only a few runners, without major differences between the groups (Table S2).

### 3.4. Urinary Creatinine, Albumin and Electrolytes Concentrations

The intake of 400-mg ibuprofen followed by running a half-marathon significantly influenced the excretion of urinary creatinine, albumin and electrolytes (Table 4). Increased excretion of potassium was accompanied by the tendency to reduce secretions of sodium and chloride, which can be explained by the activation of the renin-angiotensin-aldosterone system in order to maintain hydration status during exercise. Participants supplemented for 14 days with 200-mg MOF-VVPP prior to the run demonstrated a uniform tendency to have less prominent changes in response to this ibuprofen/exercise challenge. For example, the mean post/pre difference for creatinine was 5.22 mmol/L (95% CI: 2.46–7.98) in the MOF-VVPP group and 6.55 mmol/L (95% CI: 2.72–10.38) in the placebo group. Mean post/pre difference for albumin was 68.7 mmol/L (95% CI: 35.2–102.3) and 99.6 mmol/L (95% CI: 47.4–151.8) in the MOF-VVPP and the placebo groups, respectively. The albumin/creatinine ratio also tended to increase less in the group supplemented with MOF-VVPP (Table 4). 

### 3.5. Biomarkers of Renal Inflammation and Oxidative Stress

**Figure 3:**
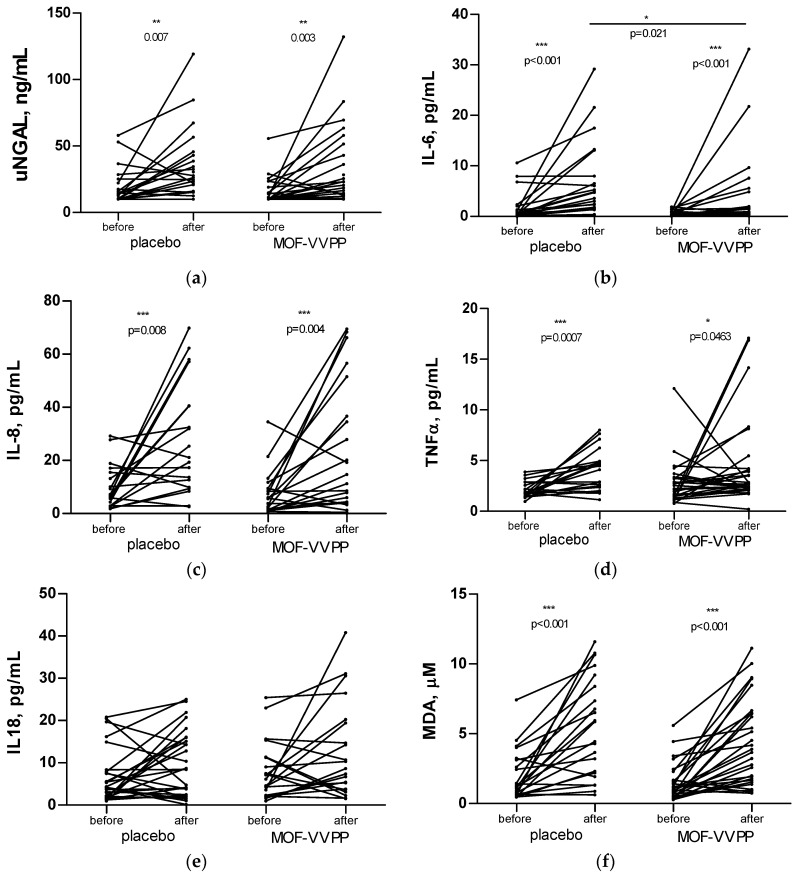

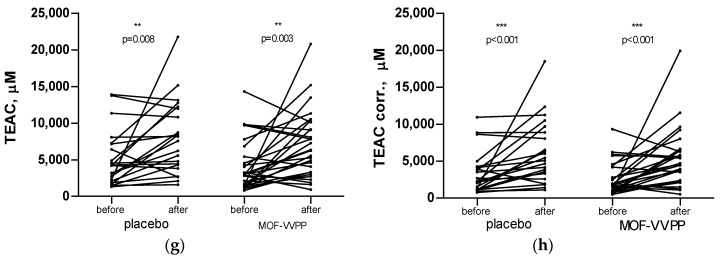
Changes in urinary neutrophil gelatinase-associated lipocalin, uNGAL (**a**), interleukin (IL)-6 (**b**), IL-8 (**c**), tumor necrosis factor (TNF)-α (**d**), IL-18 (**e**), malondialdehyde, MDA (**f**), trolox-equivalent antioxidant capacity, TEAC (**g**) and TEAC corrected for uric acid TEACcorr. (**h**) in the urine. * *p* < 0.05, ** *p* < 0.01, *** *p* < 0.001. Data are presented in absolute values as symbols and lines. Urine samples were obtained before and after the use of a 400-mg single-dose ibuprofen followed by the completion of the half-marathon. Within-group changes were assessed by the Wilcoxon matched-pairs signed rank test, and between-group changes were tested by the Mann-Whitney U test.

The prerace concentration of uNGAL did not differ significantly between the groups (Table S3). In 11 (44%) participants of the placebo group and in 16 (55%) runners of the MOF-VVPP group, prerace values of uNGAL were <10 ng/mL. Intake of a 400-mg single-dose ibuprofen followed by the completion of the half-marathon caused a significant increase in uNGAL both in the placebo and the MOF-VVPP groups (Figure 3). Supplementation with MOF-VVPP for 14 days tended to reduce the magnitude of the uNGAL increase after the ibuprofen/half-marathon challenge, with an absolute increase of 14.4 ng/mL (95% CI: 3.6–25.2) in the verum group vs. 17.0 ng/mL (95% CI: 6.2–27.8) in the placebo group (*p* = 0.688). Interestingly, after the race, four (16%) runners from the placebo and seven (24%) runners from the MOF-VVPP groups had uNGAL of <10 ng/mL (*p* = 0.735). 

Cystatin C is a novel kidney injury biomarkers used in clinical practice that is independent of age, gender and muscle mass [31]. Running already of 10 km was shown to cause an increase in urinary cystatin C concentrations up to 118 ng/mL [6]. In the present study, where the detection limit of cystatin C was 0.30 mg/L, urinary cystatin C was found in one runner of the placebo group and one runner of the MOF-VVPP group before the ibuprofen/half-marathon challenge. In the postrace urine samples, two runners in each group showed measurable levels of cystatin C (data not shown). In all cases, the concentration of cystatin C did not increase more than two-fold, and this increase was not regarded as clinically significant. Similarly, some increases in another biomarker of kidney injury beta-2-microglobulin was noted in one runner from each group before the ibuprofen/half-marathon challenge and in two runners in the placebo and three participants from the MOF-VVPP group after the race. In the mentioned cases, the concentrations of beta-2-microglobulin were just above the detection limit of 0.2 mg/L and did not exceed 0.53 mg/L. 

Participation in the half-marathon preceded by the intake of a single low dose of ibuprofen was accompanied by a statistically significant increase in proinflammatory IL-6 concentrations in urine (T = 247, z = 3.912, *p* < 0.001, r = 0.83). Importantly, this increase was largely prevented by the supplementation with MOF-VVPP for 14 days before the challenge (U = 209, z = −2.319, *p* = 0.032, r = -0.324; Figure 3 and Table S3). Participants from the placebo group demonstrated a significant increase in urinary concentrations of IL-8 (T = 162, z = 2.696, *p* = 0.007, r = 0.62) and TNF-α (T = 236, z = 3.555, *p* < 0.001, r = 0.76), which tended to be reduced by the MOF-VVPP supplementation (IL-8: U = 229, z = −1.544, *p* = 0.123, r = −0.22 and TNF-α: U = 249, z = −1.373, *p* = 0.17, r = −0.19). The concentration of IL-18 tended to rise after the ibuprofen/half-marathon challenge, both in the placebo and the MOF-VVPP groups. The increase in both groups did not reach statistical significance, and the values after the run did not differ between the groups (Table S3). 

The intake of ibuprofen followed by the completion of the half-marathon was associated with an increase in the oxidative stress biomarker MDA and with the upregulation of the antioxidant defense, revealed by an increase in urinary TEAC values (Figure 3 and Table S3). In the group of runners supplemented for 14 days with MOF-VVPP before the ibuprofen/half-marathon challenge, the tendency to a less prominent increase in urinary MDA was noted, with a mean increase of 2.97 ± 2.80 μM vs. 3.29 ± 3.23 μM in the placebo group (*p* = 0.324). The total antioxidant capacity (TEAC) of urine increased by two point one-fold in participants from the MOF-VVFF group and by one point eight-fold in runners from the placebo group. When corrected for the urinary uric acid concentrations, the total antioxidant capacity was shown to increase two point six-fold in the MOF-VVPP and two point three-fold in the placebo group. This points to the tendency of an improved antioxidant response in runners who received supplementation with this flavanolic blend (Figure 3 and Table S3). 

### 3.6. Safety 

In general, the intake of MOF-VVPP was well tolerated, with only one participant reporting mild nausea and diarrhea upon start of the supplementation, which resolved spontaneously within 48 h. Upon the intake of 400 mg of ibuprofen followed by the completion of the half-marathon, three runners of the MOF-VVPP group and two runners of the placebo group had mild-to-moderate gastrointestinal complaints, which resolved spontaneously within 48 h. 

## 4. Discussion

The present study demonstrated that the intake of a 400-mg single dose of ibuprofen followed by running a half-marathon caused some degree of renal dysfunction, as suggested by an increase in urinary creatinine, albumin, NGAL and the development of hematuria in recreational runners. The reversible decline in the kidney function after strenuous exercise is a well-recognized phenomenon in sports physiology [32]. Hemodynamic alterations cause changes in the glomerular permeability and trigger tubular dysfunction, which are primarily responsible for an increase in kidney injury biomarkers in runners [32]. Moreover, enhanced production of the proinflammatory cytokines and pro-oxidants associated with running may contribute to the transient strain on many organs of the human body, including the kidneys [33,34]. 

The intake of ibuprofen is also a known risk factor for kidney injury in runners due to its adverse effect on the glomerular perfusion [35]. Despite the fact that ibuprofen is an anti-inflammatory drug, in the setting of strenuous exercise, it has been shown to promote systemic inflammation and endotoxemia, as shown by an increase in lipopolysaccharide in blood [36]. In a randomized clinical trial evaluating the effects of ibuprofen (400 mg taken every 4 h) during an 80-km ultramarathon, an increase in the rates of AKI was reported, although this effect was, by a small margin, not statistically inferior to the placebo [37]. Longer distances and high doses of NSAIDs are generally related to a higher probability of developing kidney damage in athletes [5,38]. Despite accumulating evidence on the potential harmful effects of NSAIDs and the absence of studies that convincingly prove significant exercise performance benefits from taking NSAIDs [39,40], many runners still use them, with various motivations. Older age, female gender, competing on a longer distance, history of injuries and exercise-associated muscle pains were associated with a higher probability to take NSAIDs before/during competition by long-distance runners [12]. With or without the use of NSAID, there is no evidence that practicing a sport can lead to the development of chronic kidney disease [35,41].

In the present study, we used a single low-dose (400 mg) ibuprofen followed by running a half-marathon as a “model” of transient kidney dysfunction. This “model” not only allows to study the effects of real-world race conditions on the kidney functions of recreational athletes but also has great potential to investigate the subtle effects of dietary compounds on the adaptive responses to physiological stress, as well as the interaction between drugs and nutrients in physically active individuals. In the present study, we demonstrated that the ibuprofen/half-marathon challenge can increase urinary NGAL concentrations, as well as the albumin/creatinine ratio. Moreover, it can change the excretion of urinary electrolytes and increase concentrations of biomarkers of inflammation and oxidative stress in the urine. 

The composition of a diet can indeed be an important protective factor against exercise-induced organ dysfunction. In a recent study by Mielgo-Ayuso et al., it was shown that a diet and specific dietary products may play an important role in the optimization of the beneficial effects of exercise and may help to limit the possible adverse effects related to strenuous physical activity [42]. The consumption of meat, butter and fatty meat by runners was linked to an increased probability of developing exercise-induced cardiac stress and muscle damage after an ultramarathon, while eating fish, vegetables and olive oil was shown to be protective against the abovementioned organ damage [42]. Except for adequate hydration, no other nutritional strategies have been explored so far to exert protective effects against exercise-induced kidney dysfunction.

In the present study, the effects of a 14-day dietary supplementation with a well-characterized and standardized flavanolic blend derived from grape seeds and pine bark was investigated. Flavanols represent one of the most abundant classes of flavonoids. They can be found in a wide variety of vegetables and plant-derived food products, including wine, fruit juices, tea leaves, cocoa beans, fruits, cereal grains and legume seeds [43]. The potential beneficial effects of flavanols from various sources, including New Zealand blackcurrants, pomegranates and cherry extracts, were studied in exercise settings [26,44]. They were linked to improvements in exercise performances due to their pleiotropic actions, which involved antioxidant and anti-inflammatory, as well as protective, effects on the vascular endothelium [24,26,44]. Flavanols were also linked to preserving renal function and preventing renal diseases, although this evidence is largely derived from animal studies [45]. The renoprotective actions of grape seed extracts rich in proanthocyanidins administered for 22 weeks to spontaneously hypertensive rats were attributed to the dose-dependent reduction in albuminuria and an inflammatory infiltration at the renal interstitium [46]. To the best of our knowledge, there are no studies available in the literature that have investigated the effects of flavanols on the kidney functions in recreational runners.

In the present research, we demonstrated that the supplementation with the flavanolic blend of a grape seed and pine bark extract prevents an increase of kidney injury biomarkers related to the ibuprofen/half-marathon challenge. Indeed, tendencies to less prominent increases in urinary NGAL creatine, albumin and a lower albumin/creatinine ratio were noted after 14-day MOF-VVPP supplementations compared to the placebo. NGAL is a neutrophil-derived protein which, in the setting of kidney damage, is largely produced by the renal tubular cells in the loop of Henle and collecting ducts of the nephron. Increased concentrations of uNGAL have been linked to tubular damage and are regarded as reliable biomarkers of AKI both in clinical and exercise settings [47,48,49]. The protective effects of MOF-VVPP at the glomerular level can be suggested from the tendency to decrease proteinuria and the albumin/creatinine ratio, as well as from the significant reduction of the number of cases of exercise-related hematuria compared to the placebo. The appearance of blood in the urine after strenuous exercise reveals an increased glomerular permeability to erythrocytes. Several mechanisms have been postulated to be involved in this phenomenon, including vascular spasms, ischemia, endothelial dysfunction and oxidative stress [50]. The investigated MOF-VVPP appears to act on both the tubular and glomerular sites of possibly exercise-related alterations in the kidney functions. The less frequent and less severe hematuria upon MOF-VVPP supplementation can be attributed to its anti-inflammatory action [24,25], which was also shown in this study by a significant reduction of the IL-6 concentrations and the tendency to limit oxidative stress and potentiate an antioxidant defense during/after exercise. 

The limitations of the study include the relatively small sample size and variability in the training status and body composition between the recreational runners. A more detailed definition of the inclusion/exclusion criteria may allow to reduce the inter-person variability in the uNGAL response to the ibuprofen/exercise challenge. The effects of the flavanolic supplementation on the uNGAL concentrations could have been underestimated due to the fact that the lower limit of detection for uNGAL in this study was 10 ng/mL. 

## 5. Conclusions

Running a half-marathon is a common ambition of recreational athletes. Many runners use NSAID before/during the competition, which additionally strains the kidney functions. Results of this study showed that dietary supplementations with a well-characterized and standardized blend of monomeric and oligomeric flavanols ameliorates the renal dysfunction and attenuates inflammation and oxidative stress induced by the ibuprofen/half-marathon challenge. In general, the results of this study point to the nutritional strategies that might be useful to reduce the side effects related to use of NSAIDs and exercise. Moreover, they can facilitate the further development of a human stress model, which can be applied for testing various nutritional interventions. 

## Figures and Tables

**Table 1:** Composition of test capsules containing 50:50 blend of extracts isolated from grape seeds (*Vitis vinifera* L.) and pine bark (*Pinus pinaster* L.). n.a., not applicable.

Compound	*Vitis vinifera* L. extract% (m/m)	*Pinus pinaster* L. extract% (m/m)
Total polyphenols	90–95	ca. 80
Flavan-3-ols	60–70	n.a.
Other polyphenols	20–35	n.a.
Monomers	25–30	7–20
Oligomeric proanthocyanidins (2–5 units)	35–40	19–38
Dimeric proanthocyanidins	30–35	11–22
Polymers (>6 units)	1–5	4–13
Taxifolin	n.a.	<3
Water	ca. 5	ca. 4

**Table 2:** General characteristics of the study participants.

Characteristics	Placebo, *n =* 25	MOF-VVPP, *n* = 29	Total, *n =* 54	*p ^#^*
Male/female, *n*	15/10	18/11	33/21	0.885
Age, y	47 ± 15	47 ± 11	47 13	0.823
Blood pressure, mm Hg				
Systolic	131 ± 14	132 ± 17	131 ± 15	0.755
Diastolic	82 ± 7	83 ± 11	83 ± 9	0.807
HR, bmp	59 ± 9	58 ± 8	59 ± 8	0.695
BMI, kg/m^2^	24.8 ± 2.5	24.4 ± 2.1	24.6 ± 2.3	0.557
Average training for 3 months before half-marathon, km/week	30 (25–45)	35 (25–48)	30 (25–45)	0,848
Long-distance running experience, years	10 (5–15)	9 (5–14)	9 (5–15)	0.721
Previous races, *n*				
42.2 km	0(0–3)	1 (0–4)	0 (0–4)	0.722
21.1 km	5.0 (1–12) *	6 (3–15)	5.5 (2.8–15)	0.423
10 km	10 (4–20)	10 (4–17.5)	10 (4–20)	0.869
5 km	4.5 (0.3–10)	1 (0–10)	3 (0–10)	0.266
Reported best 21.1-km finishing time, min	110 (101–117)	110 (96.5–127.5)	110 (98–121)	0.889

Gender distribution is given in absolute numbers; all other parameters are presented as median (range) or mean ± SD. HR—heart rate, bpm—beats per minute, BMI—body mass index and MOF-VVPP—monomeric and oligomeric flavanols isolated from *Vitis vinifera* L. seeds and *Pinus pinaster* L. bark; * 4 runners in the placebo group had no experience in completing a half-marathon. *^#^* Statistical differences between the test groups were assessed by the exact Fisher’s test, unpaired *t*-test or Mann-Whitney U test. During the comparison of the prerace values between the groups, no significant differences were noted.

**Table 3:** Clinical and performance characteristics in recreational runners before and after the use of a 400-mg single dose of ibuprofen followed by the completion of the half-marathon.

Characteristics	Placebo, *n =* 25		MOF-VVPP, *n =* 29		*p ^#^*
	Before	After	Before	After	
Body weight, kg	74.7 ± 10.7	74.0 ± 10.7 *	74.8 ± 11.2	74.2 ± 11.1 *	0.935
Change of body weight, %	0.87 ± 0.69		0.77 ± 0.84		0.599
Finishing time, min					
Male	122.4 ± 11.2		117.4 ± 19.5		0.407
Female	122.9 ± 13.8		124.0 ± 19.2		0.882
Tiredness, AU	4.25 ± 2.52		4.89 ± 2.89		0.380
Muscle pain, AU	2.46 ± 3.11		2.97 ± 3.04		0.552

Body weight was assessed before and after the use of a 400-mg single-dose ibuprofen followed by completion of the half-marathon; tiredness and muscle pain were evaluated only after this challenge. Runners were supplemented with MOF-VVPP (200 mg/d) or placebo for 14 days preceding the measurements and the ibuprofen/half-marathon challenge. *—significant difference (*p <* 0.0001) for the comparison “before” vs. “after” values within the group assessed by a paired Student’s *t*-test; #—denotes the p-value for the comparison of the “after” values between both study groups by an unpaired Student *t*-test. AU—arbitrary units on the scale from 0 to 10 and MOF-VVPP—monomeric and oligomeric flavanols isolated from *Vitis vinifera* L. seeds and *Pinus pinaster* L. bark.

**Table 4:** Conventional urinary biochemistry parameters in recreational runners before and after the use of a 400-mg single-dose ibuprofen followed by the completion of the half-marathon.

Parameter	Placebo		MOF-VVPP		p *
	Before	After	Before	After	
Specific gravity, kg/L	1.007 (1.005–1.018)	1.015 ^b^ (1.006–1.024)	1.006 (1.005–1.012)	1.013 ^b^ (1.007–1.019)	0.573
Osmolality, mOsm/kg	314 (207–704)	407 (226–657)	258 (164–485)	374 (219–569)	0.490
Creatinine, mmol/L	4.0 (2.4–7.8)	10.7 ^c^ (6.3–18.0)	3.6 (1.8–6.6)	8.5 ^a^ (5.1–15.9)	0.574
Albumin **, mg/L	5.0 (5.0–5.0)	51.5 ^c^ (35.0–198.0)	5.0 (5.0–5.0)	39.0 ^c^ (13.8–105.3)	0.335
Albumin/Creatinine, mg/mmol	0.47 (0.34–0.79)	6.90 ^c^ (2.35–11.25)	0.51 (0.35–1.02)	4.2 ^c^ (2.2–10.7)	0.534
Urea, mmol/L	131 (82–306)	160 (98–225)	121 (62–246)	126 (88–281)	0.842
Sodium, mmol/L	55.5 (28.0–121.8)	50.0 (26.5–81.0)	44.0 (25.0–72.1)	34.5 (24.0–56.3)	0.130
Potassium, mmol/L	36.1 (27.5–58.4)	63.3 ^a^ (35.0–93.8)	30.5 (16.1–54.0)	53.1 ^a^ (29.8–86.2)	0.475
Chloride, mmol/L	68.5 (39.3–138.3)	40.0 ^b^ (30.0–90.0)	55.0 (34.0–86.5)	52.5 (35.3–72.8)	0.762
Uric acid, mmol/L	0.91 (0.56–1.96)	1.47 (0.64–2.20)	0.95 (0.48–1.47)	1.19 (0.56–2.00)	0.486

Data are presented as median and (25–75% percentiles). Urinary samples were obtained before and after the use of a 400-mg single-dose ibuprofen followed by the completion of the half-marathon. Runners were supplemented with MOF-VVPP (200 mg/d) or placebo for 14 days preceding urine sampling and the ibuprofen/half-marathon challenge. Statistically significant differences within the groups were assessed by the Wilcoxon matched-pairs signed rank test, ^a^ *p <* 0.05, ^b^ *p <* 0.01 and ^c^ *p <* 0.001. Statistically significant differences between the test groups were assessed by the Mann-Whitney U test. During the comparison of the “before” values between the groups, no significant differences were noted. *—denotes the p-value for the comparison of the “after” values between the groups. ** Albumin before the run was detected in 3 runners from the placebo group and 4 runners from the MOF-VVPP group. MOF-VVPP—monomeric and oligomeric flavanols isolated from *Vitis vinifera* L. seeds and *Pinus pinaster* L. bark.

## Supplementary Materials

The following are available online at https://www.mdpi.com/2072-6643/12/6/1634/s1. Table S1: Blood pressure and heart rate in recreational runners before and after the use of 400-mg single-dose ibuprofen followed by completion of the half-marathon. Table S2: Parameters assessed with the urine dipstick test in recreational runners before and after the use of 400-mg single-dose ibuprofen followed by completion of the half-marathon. Table S3: Urinary biomarkers in recreational runners before and after the use of 400-mg single-dose ibuprofen followed by completion of the half-marathon.
