# Supplementary material for: Effects of Monomeric and Oligomeric Flavanols on Kidney Function, Inflammation and Oxidative Stress in Runners: A Randomized Double-Blind Pilot Study
Source: Nutrients. 2020 Jun 1;12(6):1634. doi: 10.3390/nu12061634 (PMC7353060; doi:10.3390/nu12061634)
Supplement: Supplementary file 1 [file nutrients-12-01634-s001.pdf]

nutrients

Table S1. Blood pressure and heart rate in recreational runners before and after use of 400-mg single-dose ibuprofen followed by completion of the half-marathon

**Characteristics**	**Placebo, n=25**		*p**	**MOF-VVPP, n=29**		*p**	*p#*
	Before	*After*		*Before*	*After**		
Blood pressure, mm Hg							
Systolic	142±17	116±13	*<0.0001*	148±25	117±14	*<0.0001*	*0.801*
Diastolic	84±12	68±9	*<0.0001*	86±12	67±9	*<0.0001*	*0.928*
HR, bmp	112±14	97±11	*0.0005*	111±16	95±16	*0.0007*	*0.632*

Blood pressure and heart rate were assessed before and after the use of 400 mg single-dose ibuprofen followed by completion of the half-marathon. Runners were supplemented with MOF-VVPP (200 mg/d) or placebo for 14 days preceding the measurements and the ibuprofen/half-marathon challenge. * - denoted the p values for the comparison "before" versus "after" values within the group by paired t-test; # - denotes the p value for the comparison of the "after" values between both study groups by unpaired t-test. Bpm – beats per minute, HR – heart rate, MOF-VVPP – monomeric and oligomeric flavanols isolated from *Vitis vinifera L.* seeds and *Pinus pinaster L.* bark.

Table S2. Parameters assessed with the urine dipstick test in recreational runners before and after use of 400-mg single-dose ibuprofen followed by completion of the half-marathon

**Parameter**	**Placebo, n=25**		**MOF-VVPP, n=29**		*p**
	Before	*After*	*Before*	*After*	
Leukocytes					
2+	-	-	1 (3.4)	-	*1.000*
Urobilinogen					
1+	-	2 (8.0)	1 (3.4)	1 (3.4)	*0.535*
2+	-	1 (4.0)	-	1 (3.4)	
Blood					
0.5+	4 (16.0)	11 (44.0)	6 (20.4)	8 (27.2)	
1+	-	1 (4.0)	-	2 (6.8)	*0.110*
2+	1 (4.0)	2 (8.0)	-	1 (3.7)	
3+	-	2 (8.0)	-	-	
Bilirubin					
1+	3 (12.0)	4 (16.0)	3 (10.2)	4 (13.6)	*0.822*
Ketones					
1+	-	4 (16.0)	-	-	*0.116*
Glucose					
0.5+	-	1 (4.0)	-		
1+	-	-	-	2 (6.8)	*0.471*
2+	-	-	-	1 (3.4)	
3+	-	1 (4.0)	-	-	
Protein					
0.5+	2 (8.0)	8 (32.0)	-	12 (44.4)	
1+	-	6 (24.0)	1 (3.7)	4 (13.6)	*0.319*
2+	-	5 (20.0)	-	3 (10.2)	
3+	-	-	-	1 (3.4)	
pH					
5	15 (60.0)	15 (60.0)	21 (71.4)	22 (74.8)	
6	3 (12.0)	10 (41.7)	6 (20.4)	7 (23.8)	*0.215*
7	5 (20.0)	-	1 (3.4)	-	
8	2 (8.0)	-	-	-	
Specific gravity					
1.000	-	-	1 (3.4)	-	
1.005	3 (12.0)	1 (4.0)	6 (20.4)	3 (10.2)	
1.010	8 (32.0)	4 (16.0)	8 (27.2)	3 (10.2)	
1.015	3 (12.0)	2 (8.0)	7 (23.8)	5 (17)	*0.080*
1.020	6 (24.0)	1 (4.0)	2 (6.8)	7 (23.8)	
1.025	1 (4.0)	4 (16.0)	1 (3.4)	3 (10.2)	
1.030	4 (16.0)	13 (54.0)	4 (13.6)	6 (20.4)	
>1.030	-	1 (4.0)	-	1 (3.4)	

Data presented as absolute numbers (percentage) per group. Testing was performed in urine samples obtained before and after the use of 400 mg single-dose ibuprofen followed by completion of the half-marathon. Runners were supplemented with MOF-VVPP (200 mg/d) or placebo for 14 days preceding urine sampling and the ibuprofen/half-marathon challenge. * - denotes the p value for the comparison of the differences between the groups by Mann-Whitney U test. Nitrite was not found in any of the samples. MOF-VVPP – monomeric and oligomeric flavanols isolated from *Vitis vinifera L.* seeds and *Pinus pinaster L.* bark.

Table S3. Urinary biomarkers in recreational runners before and after use of 400-mg single-dose ibuprofen followed by completion of the half-marathon

**Urinary biomarker**	**Placebo**		**MOF-VVPP**		*p**
	Before	*After*	*Before*	*After*	
uNGAL, pg/mL	12.8 [10.0-22.2]	25.5[a] [15.3-42.0]	10.0 [10.0-14.9]	16.5 [10.1-39.7]	*0.156*
IL-6, pg/mL	0.35 [0.15-1.24]	3.57[a] [1.56-8.00]	0.25 [0.13-0.76]	1.46[a] [0.54-4.15]	***0.032***
IL-8, pg/mL	7.09 [2.88-16.31]	20.37[b] [9.75-40.51]	5.52 [1.25-9.44]	8.22[b] [3.22-36.14]	*0.125*
TNFα, pg/mL	1.85 [1.55-2.81]	4.59[a] [2.51-6.25]	2.14 [1.28-3.23]	2.74[c] [2.19-4.18]	*0.173*
IL-18, mg/mL	6.62 [2.25-11.34]	8.00 [3.77-19.63]	4.15 [1.97-7.80]	8.51 [2.38-15.75]	*0.406*
MDA, µM	1.23 [0.60-3.00]	5.85[a] [2.19-8.39]	0.95 [0.44-1.62]	3.78[a] [1.69-6.47]	*0.260*
TEAC, µM	4206 [1933-6973]	7675[b] [4290-11999]	2946 [1520-4978]	6290[b] [3294-9890]	*0.489*
TEAC corr., µM	2269 [1149-4093]	5344[a] [3313-8872]	1814 [891-3490]	4639[a] [2354-6379]	*0.441*

Data are presented as median and [25% - 75% percentiles]. Testing was performed in urine samples obtained before and after the use of 400 mg single-dose ibuprofen followed by completion of the half-marathon. Runners were supplemented with MOF-VVPP (200 mg/d) or placebo for 14 days preceding urine sampling and the ibuprofen/half-marathon challenge. Statistical significant differences within the groups were assessed by the Wilcoxon matched-pairs signed rank test, [a]$p<0.05$, [b]$p<0.01$, [c]$p<0.001$. Statistically significant differences between the test groups were assessed by the Mann-Whitney U test. During the comparison of the "before" values between the groups no significant differences were noted. * - denotes the p value for the comparison of the "after" values. IL – interleukin, MDA – malonic dialdehyde, MOF-VVPP - monomeric and oligomeric flavanols isolated from *Vitis vinifera L.* seeds and *Pinus pinaster L.* bark, TEAC – trolox equivalent antioxidant capacity, TEAC corr. – TEAC corrected for the concentrations of uric acid, TNFα – tumor necrosis factor α, uNGAL – urinary neutrophil gelatinase-associated lipocalin.

© 2020 by the authors. Licensee MDPI, Basel, Switzerland. This article is an open access article distributed under the terms and conditions of the Creative Commons Attribution (CC BY) license (http://creativecommons.org/licenses/by/4.0/).
